# Waterborne Disease Outbreaks Associated with Splash Pads — United States, 1997–2022

**DOI:** 10.15585/mmwr.ss7308a1

**Published:** 2024-12-05

**Authors:** Hannah Lawinger, Amina Khan, Colleen Lysen, Marydale Oppert, Vince R. Hill, Jonathan S. Yoder, Virginia A. Roberts, Mia C. Mattioli, Michele C. Hlavsa

**Affiliations:** ^1^Division of Foodborne, Waterborne, and Environmental Diseases, National Center for Emerging and Zoonotic Infectious Diseases, CDC; ^2^Division of Global HIV and TB, Global Health Center, CDC; ^3^Division of Infectious Disease Readiness and Innovation, National Center for Emerging and Zoonotic Infectious Diseases, CDC; ^4^Coronavirus and Other Respiratory Viruses Division, National Center for Immunization and Respiratory Diseases, CDC

## Abstract

**Problem/Condition:**

Splash pads are recreational interactive water venues that spray or jet water on users. Splash pads are intended for children aged <5 years and designed so that water typically does not collect in areas accessible to users, thereby minimizing the risk for drowning. Splash pads were first found to be associated with waterborne disease outbreaks in 1997.

**Period Covered:**

1997–2022.

**Description of System:**

Since 1971, waterborne disease outbreaks have been voluntarily reported to CDC by state, local, and territorial health departments using a standard paper form via the Waterborne Disease and Outbreak Surveillance System (WBDOSS). Beginning in 2009, WBDOSS reporting was made available exclusively through the National Outbreak Reporting System, a web-based platform. This report characterizes waterborne disease outbreaks associated with splash pads reported to CDC that occurred during 1997–2022.

**Results:**

During 1997–2022, public health officials from 23 states and Puerto Rico reported 60 waterborne disease outbreaks associated with splash pads. These reported outbreaks resulted in 10,611 cases, 152 hospitalizations, 99 emergency department visits, and no reported deaths. The 40 (67%) outbreaks confirmed to be caused, in part, by *Cryptosporidium* resulted in 9,622 (91%) cases and 123 (81%) hospitalizations. Two outbreaks suspected to be caused by norovirus resulted in 72 (73%) emergency department visits.

**Interpretation:**

Waterborne pathogens that cause acute gastrointestinal illness can be transmitted by ingesting water contaminated with feces from infected persons. Chlorine is the primary barrier to pathogen transmission in splash pad water. However, *Cryptosporidium* is tolerant to chlorine and is the most common cause of reported waterborne disease outbreaks associated with splash pads.

**Public Health Action:**

Public health officials and the aquatics sector can use the findings in this report to promote the prevention of splash pad–associated outbreaks (e.g., recommended user behaviors) and guide the construction, operation, and management of splash pads. Public health practitioners and the aquatics sector also can collaborate to voluntarily adopt CDC’s Model Aquatic Health Code recommendations to prevent waterborne illness associated with splash pads.

## Introduction

Splash pads, also known as water playgrounds, interactive fountains, spray pads, spray parks, and wet decks, spray or jet water on users. Water can either be recirculated or pass once through the venue plumbing. In recirculating splash pads, after being sprayed or jetted, the water drains into a tank and is filtered and disinfected before being sprayed or jetted again. In single-pass splash pads, water circulates through the plumbing only once before draining, typically into a sewer system (*1*).

Splash pads, which first appeared in the 1990s, are designed so that water typically does not collect in areas accessible to users (*1*). Although this feature minimizes the risk for drowning, splash pads have been associated with waterborne outbreaks of either infectious or chemical etiology. Because splash pads do not have standing water in areas accessible to users, they might be exempt from public health regulations in certain jurisdictions. Only 13 states regulated splash pads before 2000 (*2*). Additional states (e.g., Oregon) have since enacted public health regulations for splash pads, often in response to splash pad–associated outbreaks (*2*).

Public health officials investigate waterborne disease outbreaks associated with splash pads and can voluntarily report epidemiologic, laboratory, and environmental health data collected during investigations to CDC. This report summarizes data on splash pad–associated outbreaks reported to CDC from 1997, the year of the first two reported splash pad–associated outbreaks (*3*,*4*), through 2022, the most recent year for which data are available. Although splash pad–associated outbreaks have been previously reported individually (*5*) or as part of a summary on waterborne disease outbreaks (*6*), this report is the first summary of waterborne disease outbreaks associated with splash pads. Public health officials and the aquatics sector can use the findings in this report to promote the prevention of splash pad–associated outbreaks (e.g., recommended user behaviors) and guide the construction, operation, and management of splash pads.

## Methods

### Data Sources

In 1971, CDC established the Waterborne Disease and Outbreak Surveillance System (WBDOSS) to systematically collect data on waterborne disease outbreaks via paper forms. WBDOSS did not officially collect data on outbreaks associated with recreational water (e.g., pools and lakes) until 1978. Jurisdictions (i.e., the 50 states, the District of Columbia, U.S. territories, and freely associated states) voluntarily report data on recreational waterborne disease outbreaks. Until 2009, jurisdictions voluntarily reported outbreaks to CDC using a standard paper form.

In 2009, CDC launched the National Outbreak Reporting System (NORS), a web-based platform designed to enable voluntary electronic reporting of data on waterborne and other disease outbreaks (i.e., foodborne, enteric animal contact, environmental, and indeterminate or unknown modes of transmission) (https://www.cdc.gov/nors/about/index.html). Since 2009, all waterborne outbreaks are reported exclusively through NORS. NORS Form 52.12 that was used to report waterborne outbreaks through 2022 does not represent the version used to launch NORS in 2009 because changes were made post-launch (https://www.cdc.gov/nors/pdf/NORS_CDC_5212-form.pdf). All data from WBDOSS were merged into NORS after NORS launched. In 2023, CDC substantially updated NORS, including revising and streamlining the collection of data on waterborne disease outbreaks (Form 52.14) (https://www.cdc.gov/nors/pdf/NORS-Form-CDC-52.14_508.pdf). CDC annually requests data on waterborne disease outbreaks from jurisdictions and coordinates with health departments to clean and finalize the data.

### Definitions

Recreational water can be classified as treated or untreated. Treated recreational water undergoes systematic treatment (e.g., disinfection or filtration) to maintain quality for recreational use and is typically in an enclosed and manufactured structure. In addition to pools and hot tubs, splash pads are treated recreational water venues. Splash pads can include single-pass splash pads in which treated tap water is circulated but does not undergo additional treatment within the splash pad. Untreated recreational water does not undergo systematic treatment to maintain quality for recreational use. Lakes, rivers, and oceans are untreated recreational water venues.

A recreational waterborne disease outbreak is the occurrence of similar illness (e.g., gastrointestinal or respiratory) in two or more persons epidemiologically linked by location and time of exposure to recreational water or pathogens or chemicals aerosolized or volatilized into the air surrounding the water. Recreational waterborne disease outbreaks associated with at least one splash pad can be classified as splash pad–associated outbreaks.

### Variables

Data summarized for each outbreak include exposure jurisdiction (the NORS variable that collects data on jurisdiction of outbreak exposure is the exposure state); earliest illness onset date; etiology (confirmed or suspected); venue or venues (at least one splash pad implicated as the outbreak source); setting (the location of the implicated venue or venues [e.g., community or municipal park or zoo]); contributing factors; and counts of cases of illness, emergency department visits, hospitalizations, and deaths. These data were consistently collected during 1997–2022, except for counts of emergency department visits, which have been collected since 2009 with the launch of NORS. Outbreaks with multiple etiologies were classified and analyzed as one outbreak.

The setting refers to where the exposure to water occurred. Examples of water settings include community or municipality, community or municipal park, water park, recreational facility, and zoo. In NORS, a community or municipality setting is defined as a city, town, or other settlement where a large group of persons live and work. A community or municipal park setting is defined as a park owned by a community or municipality. The water venue refers to the type of venue located in the water setting. Examples of water venues include splash pads, swimming pools, and hot tubs.

This report also summarizes NORS data for factors that contribute to outbreaks (i.e., contributing factors). Jurisdictions can report contributing factors in NORS using a list of up to 41 factors specific to treated recreational waterborne disease outbreaks or enter additional contributing factors in a free text field. Contributing factors can be reported as documented/observed or suspected. Contributing factors are classified as documented/observed if information is gathered during document reviews, direct observations, or interviews. Contributing factors are classified as suspected if they might have occurred but no documentation or observable evidence is available. Contributing factors for treated recreational waterborne outbreaks are categorized as being related to maintenance, persons, policy and management, and facility design.

### Analyses

This report characterizes splash pad–associated outbreaks reported and finalized in NORS as of May 1, 2024. Data analysis for each outbreak included jurisdiction; illness onset date; etiology; water venue; setting of exposure; contributing factors; and counts of cases of illnesses, emergency department visits, hospitalizations, and deaths. Data on contributing factors were analyzed for outbreaks associated with splash pads only and no other venues. To summarize data on characteristics of reported splash pad–associated outbreaks, CDC calculated descriptive statistics using R software (version 4.3.2; R Foundation). This activity was reviewed by CDC, deemed not research, and was conducted consistent with applicable Federal law and CDC policy.*

## Results

### All Splash Pad–Associated Outbreaks

**FIGURE 1:**
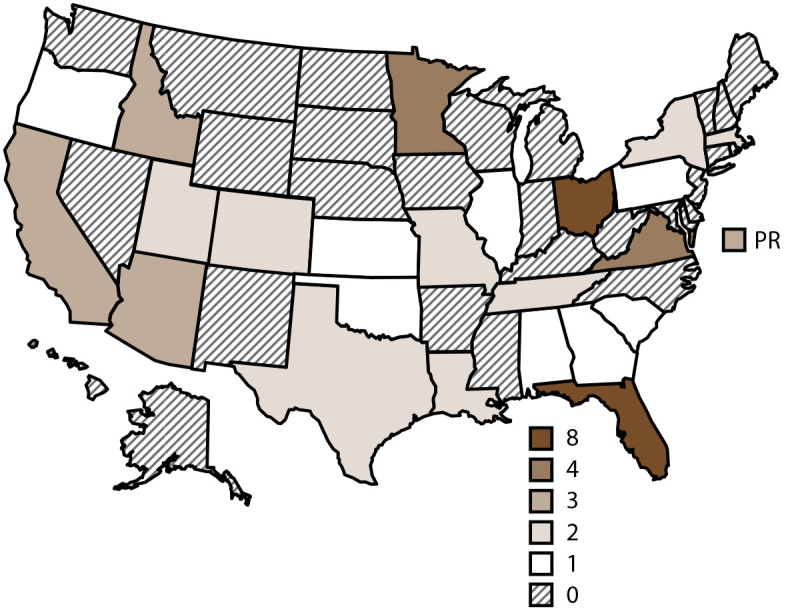
Waterborne disease outbreaks* associated with splash pads, by jurisdiction — Waterborne Disease and Outbreak Surveillance System, United States, 1997–2022 **Abbreviation:** PR = Puerto Rico. * N = 60 reported outbreaks.

During 1997–2022, public health officials from 23 states and Puerto Rico (Figure 1) reported 60 splash pad–associated outbreaks (Table 1). These 60 outbreaks were associated with exposures in settings where only splash pads were located (n = 39) as well as settings that included splash pads and other venues (e.g., swimming pools and hot tubs) (n = 21). These 60 outbreaks resulted in 10,611 cases, 152 hospitalizations, 99 emergency department visits, and no reported deaths.

**TABLE 1:** Waterborne disease outbreaks* associated with splash pads, by year, jurisdiction, and selected characteristics — Waterborne Disease and Outbreak Surveillance System, United States, 1997–2022

Year/Jurisdiction	Month	No. of cases	No. of emergency department visits^†^	No. of hospitalizations	Etiology	Venue	Setting
**1997**							
Massachusetts	July	9	—^§^	0	*Shigella sonnei*	Splash pad or waterslide	Unknown
Minnesota	June	369	—	6	*Cryptosporidium* sp.^¶^	Splash pad	Zoo
**1999**							
Florida	August	38	—	4	*Cryptosporidium parvum* and *Shigella sonnei*	Splash pad	Unknown park
**2001**							
Colorado	July	33	—	1	*Shigella sonnei*	Splash pad	Unknown
**2002**							
Massachusetts	July	767	—	—	*Cryptosporidium* sp.	Splash pad, hot tub, swimming pool, or kiddie or wading pool	Club (requires membership)
**2003**							
Oregon	July	56	—	0	*Shigella sonnei*	Splash pad	Community or municipality
**2004**							
Illinois	July	37	—	5	*Cryptosporidium* sp.	Splash pad or swimming pool	Community or municipality
**2005**							
Louisiana	August	31	—	2	*Cryptosporidium hominis*	Splash pad	Water park
New York	June	2,307	—	25	*Cryptosporidium hominis*	Splash pad	State park
**2006**							
California	July	16	—	2	*Cryptosporidium hominis*	Splash pad	Community or municipality
Florida	May	55	—	0	*Cryptosporidium hominis* and *Giardia duodenalis*	Splash pad	Community or municipality
Louisiana	July	29	—	3	*Cryptosporidium hominis*	Splash pad or swimming pool	Water park
Missouri	June	116	—	4	*Cryptosporidium hominis*	Splash pad or swimming pool	Water park
**2007**							
California	June	6	—	0	Norovirus GII	Splash pad	Community or municipality
Florida	June	25	—	—	*Cryptosporidium* sp.	Splash pad	Community or municipality
Florida	July	6	—	—	*Cryptosporidium* sp. (S)	Splash pad	Zoo
Idaho	June	31	—	—	Shiga toxin–producing *Escherichia coli* O157:H7	Splash pad	Community or municipality
Idaho	July	2,000	—	—	*Cryptosporidium hominis*	Splash pad	Community or municipality
Idaho	August	32	—	0	*Cryptosporidium hominis*	Splash pad	Community or municipality
Kansas	May	79	—	7	*Cryptosporidium* sp.	Splash pad, swimming pool, or unknown	Water park, community or municipality, private residence, or hotel, motel, lodge, or inn
Ohio	January	665	—	—	Chloramines**	Splash pad, hot tub, or swimming pool	Water park
Puerto Rico	August	107	—	3	*Cryptosporidium hominis*	Splash pad	Water park
**2008**							
Arizona	July	9	—	—	*Cryptosporidium hominis*	Splash pad	Water park
California	August	5	—	0	*Cryptosporidium* sp.	Splash pad or swimming pool	Community or municipality
Texas	June	2,050	—	5	*Cryptosporidium hominis*	Splash pad, lake, reservoir, impoundment, or swimming pool	Community or municipality
**2009**							
Puerto Rico	June	68	57	13	Norovirus (S)	Splash pad	Community or municipal park
**2010**							
South Carolina	July	31	3	0	*Cryptosporidium hominis* IbA10G2	Splash pad	Community or municipality
**2011**							
Alabama	June	19	2	3	Shiga toxin–producing *Escherichia coli* O157:H7	Splash pad, hot tub, or swimming pool	Recreational facility
New York	January	4	0	0	Chloramines (S)	Splash pad, swimming pool, or other	Water park
Ohio	July	7	0	1	*Cryptosporidium* sp.	Splash pad	Community or municipal park
Ohio	August	3	0	0	*Cryptosporidium* sp.	Splash pad	Community or municipal park
Ohio	August	4	0	1	*Cryptosporidium* sp.	Splash pad	Community or municipal park
Texas	August	130	—	—	*Cryptosporidium* sp.	Splash pad or swimming pool	Unknown
**2012**							
Colorado	June	5	0	0	*Cryptosporidium* sp.	Splash pad, swimming pool, or hot tub	Club (requires membership)
Minnesota	July	2	0	0	*Cryptosporidium parvum* IIaA16G2R2	Splash pad	Community or municipality
Minnesota	July	2	0	0	*Cryptosporidium parvum* IIaA16G2R2	Splash pad	Community or municipal park
Missouri	June	3	0	3	*Legionella pneumophila*	Splash pad, hot tub, or swimming pool	Resort
Ohio	June	16	0	0	Chlorine (S)	Splash pad, swimming pool, or public kiddie or wading pool	Recreational facility
Ohio	July	12	2	1	*Cryptosporidium* sp.	Splash pad, swimming pool, or other	Water park
Puerto Rico	December	18	15	4	Norovirus (S)	Splash pad	Community or municipal park
**2013**							
Oklahoma	May	17	1	0	Chlorine (S)	Splash pad	Unknown park
Pennsylvania	August	2	2	0	*Cryptosporidium parvum*	Splash pad	Community or municipality
**2014**							
Florida	June	19	0	0	*Cryptosporidium* sp.	Splash pad	Community or municipal park
Florida	July	6	0	0	*Shigella* sp.	Splash pad	Community or municipal park
Georgia	August	18	0	1	*Cryptosporidium hominis*	Splash pad	Community or municipal park
Tennessee	May	5	3	2	*Salmonella* serotype Newport	Splash pad	Community or municipality
**2015**							
Florida	July	7	4	0	*Cryptosporidium* sp.	Splash pad or swimming pool	Water park or child care or day care center
Virginia	May	4	0	0	*Campylobacter jejuni*	Splash pad	Community or municipal park
Virginia	July	87	2	0	*Cryptosporidium hominis* IfA12G1 and *Cryptosporidium* sp.	Splash pad, swimming pool, or private kiddie or wading pool	Camp or cabin setting
Virginia	August	2	0	0	*Cryptosporidium* sp.	Splash pad	Community or municipal park
**2016**							
Arizona	June	10	0	0	Unknown	Splash pad	Community or municipal park
Arizona	July	459	0	52	*Cryptosporidium hominis* IfA12G1	Splash pad, hot tub, temporary waterslide, swimming pool, or public and private kiddie or wading pool	Community or municipal park, public outdoor area, water park, private residence, or subdivision or neighborhood
Ohio	July	638	0	0	*Cryptosporidium hominis*	Splash pad, swimming pool, or public kiddie or wading pool	Water park or other
**2017**							
Utah	June	6	0	3	Shiga toxin–producing *Escherichia coli* O157:H7	Splash pad	Community or municipal park
**2018**							
Florida	May	7	—	—	*Cryptosporidium* sp. (S)	Splash pad	Zoo
Minnesota	July	3	0	0	*Cryptosporidium parvum* IIaA15G2R1	Splash pad	Community or municipality
**2019**							
Ohio	August	4	0	0	*Cryptosporidium* sp.	Splash pad	Community or municipal park
Virginia	July	90	4	1	*Cryptosporidium parvum* and *Cryptosporidium* sp.	Splash pad, swimming pool, or waterslide	Recreational facility
**2021**							
Tennessee	June	22	4	0	*Cryptosporidium hominis* and *Cryptosporidium parvum* (S)	Splash pad	Public outdoor area
**2022**							
Utah	June	3	0	0	*Cryptosporidium* sp.	Splash pad	Public outdoor area

**Abbreviations:** NORS = National Outbreak Reporting System; (S) = suspected etiology.

* N = 60 reported outbreaks, including 10,611 cases, 152 hospitalizations, and 99 emergency department visits.

^†^ CDC started collecting data on counts of emergency department visits with the launch of NORS in 2009.

^§^ Dashes indicate that the number is unknown.

^¶^ The etiology was reported as *Cryptosporidium* *parvum* in NORS; however, molecular diagnostic methods to distinguish *Cryptosporidium* species were not available in 1997.

** Chloramine vapor, collected via air samples, caused respiratory burns.

The outbreak etiology of waterborne disease was laboratory confirmed for 52 (87%) of the 60 outbreaks (Table 2). *Cryptosporidium* caused 40 (67%) outbreaks (including one that also was caused by *Giardia* species and another also caused by *Shigella* species) that resulted in 9,622 (91%) reported cases, 123 (81%) hospitalizations, and 21 (21%) emergency department visits. More specifically, *Cryptosporidium hominis* caused 14 (23%) outbreaks that resulted in 7,833 (74%) cases, 97 (64%) hospitalizations, and three (3%) emergency department visits. *C. hominis* also caused the three largest outbreaks (2,307 cases, 2,050 cases, and 2,000 cases); two of these outbreaks were associated with splash pads only and the remaining outbreak with a splash pad and other venues. *Cryptosporidium parvum* caused four (7%) outbreaks that resulted in nine (<1%) cases, two (2%) emergency department visits, and no hospitalizations. *Cryptosporidium* of unknown species caused 17 (28%) outbreaks that resulted in 1,478 (14%) cases, 21 (14%) hospitalizations, and six (6%) emergency department visits. Multiple species of *Cryptosporidium* caused three (5%) outbreaks that resulted in 199 (2%) cases, 10 (10%) emergency department visits, and one (1%) hospitalization.

**TABLE 2:** Waterborne disease outbreaks* associated with splash pads, by etiology — Waterborne Disease and Outbreak Surveillance System, United States, 1997–2022

Etiology	No. of outbreaks (%)^†^	No. of cases (%)^†^	Median no. of cases (minimum–maximum)
**Confirmed**	52 (87)	10,465 (99)	20.5 (2–2,307)
**Parasite**			
*Cryptosporidium* spp.	38 (63)	9,529 (90)	23.5 (2–2,307)
*Cryptosporidium hominis*	14 (23)	7,833 (74)	69.5 (9–2,307)
*Cryptosporidium parvum*	4 (7)	9 (<1)	2 (2–3)
*Cryptosporidium* unknown species	17 (28)	1,478 (14)	7 (2–767)
Multiple *Cryptosporidium* species	3 (5)	199 (2)	87 (22–90)
*Cryptosporidium hominis* and *Giardia duodenalis*	1 (2)	55 (1)	NA
**Bacterium**			
*Campylobacter jejuni*	1 (2)	4 (<1)	NA
*Escherichia coli* O157:H7	3 (5)	56 (1)	19 (6–31)
*Legionella pneumophila* ^§^	1 (2)	3 (<1)	NA
*Salmonella* serotype Newport	1 (2)	5 (<1)	NA
*Shigella* spp.	4 (7)	104 (1)	21 (6–56)
*Shigella sonnei*	3 (5)	98 (1)	33 (9–56)
*Shigella* unknown	1 (2)	6 (<1)	NA
**Virus**			
Norovirus genogroup II	1 (2)	6 (<1)	NA
**Multiple pathogen **			
*Cryptosporidium parvum* and *Shigella sonnei*	1 (2)	38 (<1)	NA
**Chemical **			
Chloramines	1 (2)	665 (6)	NA
**Suspected**	7 (12)	136 (1)	16 (4–68)
**Infectious **			
*Cryptosporidium* unknown species	2 (3)	13 (<1)	6.5 (6–7)
Norovirus	2 (3)	86 (1)	43 (18–68)
**Chemical**			
Chloramines	1 (2)	4 (<1)	NA
Chlorine	2 (3)	33 (<1)	16.5 (16–17)
**Unknown**	1 (2)	10 (<1)	NA
**Total**	**60 (100)**	**10,611 (100)**	**18 (2–2,307)**

**Abbreviation:** NA = not applicable.

* N = 60 reported outbreaks.

^†^ Percentages might not add to 100% because of rounding.

^§^ The outbreak caused by *Legionella pneumophila* was associated with multiple venues, including a hot tub.

*Shigella* caused five outbreaks (including one that was also caused by *Cryptosporidium*), *Escherichia coli* O157:H7 caused three, and the following caused one outbreak each:* Campylobacter jejuni*,* Giardia duodenalis *(also caused by* Cryptosporidium*), norovirus, and* Salmonella *serotype Newport. These 12 (20%) outbreaks resulted in 271 (3%) cases of acute gastrointestinal illness, 16 (11%) hospitalizations, and six (6%) emergency department visits. *Legionella pneumophila* and chloramines were each confirmed to cause one outbreak. Of the 99 emergency department visits reported for all splash pad–associated outbreaks, 72 (73%) resulted from two outbreaks suspected to be caused by norovirus.

**FIGURE 2:**
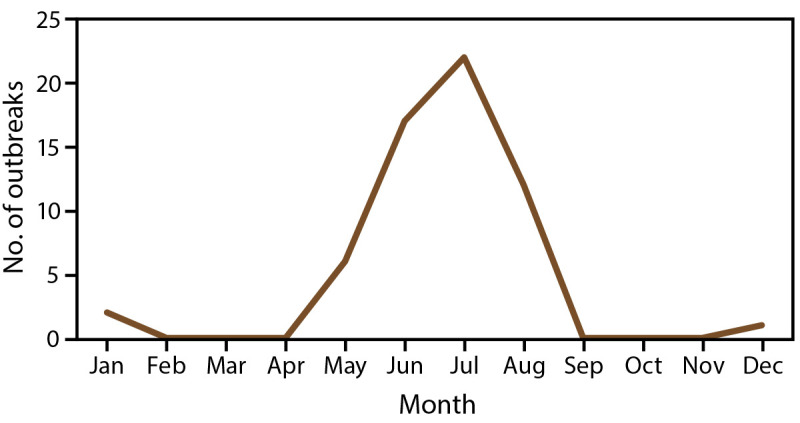
Waterborne disease outbreaks* associated with splash pads, by month — Waterborne Disease and Outbreak Surveillance System, United States, 1997–2022 * N = 60 reported outbreaks.

**FIGURE 3:**
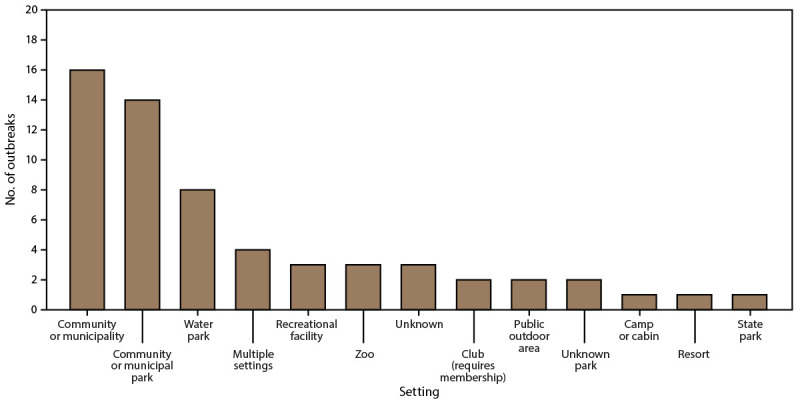
Waterborne disease outbreaks* associated with splash pads, by setting^†^ — Waterborne Disease and Outbreak Surveillance System, United States, 1997–2022 * N = 60 reported outbreaks. ^†^ Multiple settings category includes outbreaks with more than one possible setting. One outbreak each was associated with the following combinations of settings: 1) water park, or child care or day care center; 2) water park, community or municipality, private residence, or hotel, motel, lodge, or inn; 3) water park or other setting; and 4) community or municipality, public outdoor area, water park, private residence, or subdivision or neighborhood.

Of the 60 splash pad–associated outbreaks, 57 (95%) were reported during May–August (Figure 2). Approximately one fourth (n = 17; 27%) of the outbreaks were associated with at least one splash pad in a community or municipality setting, followed by 14 outbreaks (23%) in a community or municipal park (Figure 3).

### Outbreaks Associated with Splash Pads Only

Data on outbreaks associated with splash pads only were analyzed separately to evaluate only those etiologic agents and contributing factors associated with splash pads, because these might differ from outbreaks associated with other treated recreational venues (e.g., swimming pools and hot tubs). Of the 60 outbreaks, 39 (65%) were associated with splash pads only and resulted in 5,384 cases, 85 emergency department visits, and 68 hospitalizations (Table 3). An outbreak etiology was laboratory confirmed for 33 (85%) of the 39 outbreaks. *Cryptosporidium* caused 25 (64%) of the 39 outbreaks (including one that was also caused by *Giardia* and another also caused by *Shigella*) that resulted in 5,111 (95%) of 5,384 cases, nine (11%) of 85 emergency department visits, and 45 (66%) of 68 hospitalizations. More specifically, *C. hominis* caused nine (23%) outbreaks that resulted in 4,551 (85%) cases, 33 (49%) hospitalizations, and no emergency department visits. *C. parvum* caused four (10%) outbreaks that resulted in nine (<1%) cases, two (2%) emergency department visits, and no hospitalizations. *Cryptosporidium* of unknown species caused nine (23%) outbreaks that resulted in 436 (8%) cases, eight (12%) hospitalizations, and no emergency department visits. Multiple species of *Cryptosporidium* caused one (3%) outbreak that resulted in 22 (<1%) cases, four (5%) emergency department visits, and no hospitalizations.

**TABLE 3:** Waterborne disease outbreaks* associated with splash pads, by etiology and associated venues — Waterborne Disease and Outbreak Surveillance System, United States, 1997–2022

Etiology	Associated with splash pads only			Associated with splash pads and other venues		
	No. of outbreaks (%)^†^	No. of cases (%)^†^	Median no. of cases (minimum–maximum)	No. of outbreaks (%)^†^	No. of cases (%)^†^	Median no. of cases (minimum–maximum)
**Confirmed**	33 (85)	5,258 (98)	16 (2–2,307)	19 (90)	5,207 (100)	79 (3–2,050)
**Parasite**						
*Cryptosporidium* spp.	23 (59)	5,018 (93)	16 (2–2,307)	15 (71)	4,511 (86)	87 (5–2,050)
*Cryptosporidium hominis*	9 (23)	4,551 (85)	31 (9–2,307)	5 (24)	3,292 (63)	459 (29–2,050)
*Cryptosporidium parvum*	4 (10)	9 (<1)	2 (2–3)	0	0	NA
*Cryptosporidium* unknown	9 (23)	436 (8)	4 (2–369)	8 (38)	1,042 (20)	24.5 (5–767)
Multiple *Cryptosporidium* species	1 (3)	22(<1)	NA	2 (10)	177 (3)	88.5 (87–90)
*Cryptosporidium hominis* and *Giardia duodenalis*	1 (3)	55 (1)	NA	0	0	NA
**Bacterium**						
*Camplyobacter jejuni*	1 (3)	4 (<1)	NA	0	0	NA
*Escherichia coli* O157:H7	2 (5)	37 (1)	18.5 (6–31)	1 (5)	19 (<1)	NA
*Legionella pneumophila* ^§^	0	0	NA	1 (5)	3 (<1)	NA
*Salmonella* serotype Newport	1 (3)	5 (<1)	NA	0	0	NA
*Shigella* spp.	3 (8)	95 (2)	33 (6–56)	1 (5)	9 (<1)	NA
*Shigella sonnei*	2 (5)	89 (2)	44.5 (33–56)	1 (5)	9 (<1)	NA
*Shigella* unknown	1 (3)	6 (<1)	NA	0	0	NA
**Virus**						
Norovirus genogroup II	1 (3)	6 (<1)	NA	0	0	NA
**Multiple pathogen**						
*Cryptosporidium parvum* and *Shigella sonnei*	1 (3)	38 (1)	NA	0	0	NA
**Chemical**						
Chloramines	0	0	NA	1 (5)	665 (13)	NA
**Suspected**	5 (13)	116 (2)	17 (6–68)	2 (10)	20 (<1)	10 (4–16)
**Parasite, bacterium, or virus**						
*Cryptosporidium* unknown species	2 (5)	13 (<1)	6.5 (6–7)	0	0	NA
Norovirus	2 (5)	86 (2)	43 (18–68)	0	0	NA
**Chemical**						
Chloramines	0	0	NA	1 (5)	4 (<1)	NA
Chlorine	1 (3)	17 (<1)	NA	1 (5)	16 (<1)	NA
**Unknown**	1 (3)	10 (<1)	NA	0	0	NA
**Total**	**39 (100)**	**5,384 (100)**	**18 (2–2,307)**	**21 (100)**	**5,227 (100)**	**37 (3–2,050)**

**Abbreviation:** NA = not applicable.

* N = 60 reported outbreaks.

^†^ Percentages might not add to 100% because of rounding.

^§^ The outbreak caused by *Legionella pneumophila* was associated with multiple venues, including a hot tub.

*Shigella* species caused four outbreaks associated with splash pads only (including one that also was caused by *Cryptosporidium*), *E. coli* O157:H7 caused two outbreaks, and each of the following etiologic agents caused one outbreak: *C. jejuni*, *G. duodenalis* (also caused by *Cryptosporidium*), norovirus, and *Salmonella* serotype Newport (Table 3). These 10 (26%) of the 39 outbreaks resulted in 140 (3%) of 5,384 cases of acute gastrointestinal illness, 10 (15%) of 68 hospitalizations, and three (4%) of 85 emergency department visits. Of the 85 emergency department visits reported for outbreaks associated with splash pads only, 72 (85%) resulted from two outbreaks suspected to be caused by norovirus. Chlorine was suspected to cause one outbreak associated with splash pads only that resulted in 17 cases (<1%), one emergency department visit (1%), and no hospitalizations.

Of the 39 outbreaks associated with splash pads only, 38 (97%) were reported during May–August (Figure 2). Puerto Rico reported one outbreak that started in December. Of the 39 outbreaks associated with splash pads only, 14 (36%) were associated with splash pads in a community or municipal park setting, followed by 13 outbreaks (33%) associated with a splash pad in a community or municipality setting (Figure 3).

Of the 39 outbreaks associated with splash pads only, 27 (69%) had data on contributing factors reported in NORS (Table 4). Of these 27 outbreaks, a total of 70 documented/observed contributing factors were reported. For outbreaks associated with splash pads only and caused by *Cryptosporidium*, a person-related contributing factor (i.e., primary intended use of water is by diaper/toddler aged children) and a facility design–related factor (i.e., no supplemental disinfection installed that would have inactivated pathogens [e.g., *Cryptosporidium*]) were most commonly reported as documented/observed. For outbreaks associated with splash pads only that were caused by a bacterium or virus, a maintenance-related contributing factor (i.e., disinfectant control system malfunctioning, inadequate, or lacking) and a policy and management–related factor (i.e., inadequate water quality monitoring [e.g., inadequate test kit or testing frequency]) were most commonly reported. A person-related contributing factor (i.e., operator error) also was commonly reported as documented/observed for outbreaks caused by *Cryptosporidium* or by a bacterium or virus.

**TABLE 4:** Contributing factors for waterborne disease outbreaks associated with splash pad venues only,* by contributing factor category and etiology — Waterborne Disease and Outbreak Surveillance System, United States, 1999–2022

Contributing factor	*Cryptosporidium* spp.^†^ (n = 19)		Bacterial and viral pathogens (n = 6)		Chemical^§^ (n = 1)		Unknown etiology (n = 1)		Total (N = 27)	
	Documented	Suspected	Documented	Suspected	Documented	Suspected	Documented	Suspected	Documented	Suspected
**Maintenance^¶^**	5	7	21	3	0	5	0	2	**26**	**17**
Disinfectant control system malfunctioning or is inadequate or lacking (e.g., hand feed)^¶^	0	4	6	2	0	1	0	0	**6**	**7**
Incorrect settings on disinfectant control system^¶^	0	0	3	0	0	1	0	1	**3**	**2**
pH control system malfunctioning, inadequate, or lacking (e.g., hand feed)^¶^	0	0	3	0	0	1	0	0	**3**	**1**
Insufficient system checks so breakdown detection delayed^¶^	1	0	2	1	0	0	0	0	**3**	**1**
Incorrect settings on pH control system^¶^	0	0	2	0	0	1	0	1	**2**	**2**
No preventive equipment maintenance programs to reduce breakdowns^¶^	1	0	2	0	0	0	0	0	**3**	**0**
Filtration system malfunctioning or inadequate (e.g., low flow rate)^¶^	0	1	2	0	0	0	0	0	**2**	**1**
Supplemental disinfection system malfunctioning or inadequate (e.g., ultraviolet light or ozone)^¶^	1	0	1	0	0	0	0	0	**2**	**0**
Chemical handling error (e.g., chemical hookup, or improper mixing or application)^¶^	1	0	0	0	0	1	0	0	**1**	**1**
Extensive slime or biofilm formation^¶^	1	0	0	0	0	0	0	0	**1**	**0**
Recent construction^¶^	0	1	0	0	0	0	0	0	**0**	**1**
Lack of draining or cleaning^¶^	0	1	0	0	0	0	0	0	**0**	**1**
Person**	9	18	5	2	0	1	0	0	**14**	**21**
Primary intended use of water is by diaper/toddler aged children**	4	6	1	1	0	0	0	0	**5**	**7**
Operator error**	2	0	2	0	0	1	0	0	**4**	**1**
Patrons continued to swim when ill with diarrhea**	0	5	0	0	0	0	0	0	**0**	**5**
Fecal or vomitus accident**	1	4	0	0	0	0	0	0	**1**	**4**
Exceeded maximum bather load**	0	2	1	1	0	0	0	0	**1**	**3**
Heavy use by child care center groups**	2	1	1	0	0	0	0	0	**3**	**1**
**Policy and management^††^**	6	4	14	1	0	0	1	1	**21**	**6**
Inadequate water quality monitoring (e.g., inadequate test kit or testing frequency)^††^	2	2	5	1	0	0	1	0	**8**	**3**
No or inadequate policies on good chemical handling and storage practices^††^	0	0	3	0	0	0	0	0	**3**	**0**
Facility falls outside aquatic health code^††^	1	0	1	0	0	0	0	0	**2**	**0**
No operator on duty at the time of incident^††^	1	0	1	0	0	0	0	0	**2**	**0**
No shock or hyperchlorination policy^††^	2	0	0	0	0	0	0	0	**2**	**0**
Poor management^††^	0	0	1	0	0	0	0	1	**1**	**1**
Untrained or inadequately trained staff on duty^††^	0	1	1	0	0	0	0	0	**1**	**1**
Employee illness policies absent or not enforced^††^	0	0	1	0	0	0	0	0	**1**	**0**
No aquatics operators on payroll who have received state or local certified training^††^	0	0	1	0	0	0	0	0	**1**	**0**
Remote monitoring system replaces onsite water quality testing^††^	0	1	0	0	0	0	0	0	**0**	**1**
**Facility design^§§^**	6	1	1	1	0	0	0	0	**7**	**2**
No supplemental disinfection installed that would have inactivated pathogen (e.g., *Cryptosporidium*)^§§^	4	0	0	0	0	0	0	0	**4**	**0**
Hygiene facilities (e.g., toilets or diaper-changing facilities) inadequate or distant^§§^	1	1	1	1	0	0	0	0	**2**	**2**
Certain spray feature water bypasses filtration or treatment system and returns to feature unfiltered or untreated^§§^	1	0	0	0	0	0	0	0	**1**	**0**
**Unknown**	2	1	0	0	0	0	0	0	**2**	**1**
**Total**	**28**	**31**	**42**	**7**	**0**	**6**	**1**	**3**	**70**	**47**

**Abbreviation:** NORS = National Outbreak Reporting System.

* N = 27 reported outbreaks associated with splash pad venues only.

^†^ *Cryptosporidium* includes two outbreaks caused by *Cryptosporidium* and another pathogen; one outbreak was caused by *Cryptosporidium* and *Giardia* and another by *Cryptosporidium* and *Shigella*.

^§^ Chlorine burn from inhalation.

^¶^ Maintenance factor grouping on NORS form.

** The person category includes the data from people factor grouping on NORS form.

^††^ Policy and management factor grouping on NORS form.

^§§^ Facility design factor grouping on NORS form.

## Discussion

*Cryptosporidium* is the most frequently confirmed etiology of splash pad–associated outbreaks reported to CDC. This parasite, like all other laboratory-confirmed infectious etiologies in this report, except *L. pneumophila*, can be transmitted by ingesting water contaminated with feces from infected persons and cause acute gastrointestinal illness. Jurisdictions most commonly report cases of acute gastrointestinal illness in children aged <5 years, including cases caused by *Cryptosporidium* (*7*). Young children also are less likely to have mastered toileting and hygiene skills, and swim diapers do not prevent fecal contamination of recreational water (*8*). Sitting or standing on top of water jets and wearing diapers or swim diapers are behaviors commonly observed in children playing in splash pads (*9*,*10*). The former behavior results in rinsing of diapers or perianal surfaces, which in young children can carry as much as 10 g of feces (*11*). Thus, because of their design, splash pads can be at increased risk for contamination with pathogens. Because children typically ingest more recreational water than adults (*12*) and have been commonly observed to place their open mouths on sprayed or jetted water (*9*,*10*), children are at increased risk for exposure to pathogens in contaminated splash pad water.

Chlorine, a chemical disinfectant, is the primary barrier to pathogen transmission in treated recreational water. Most pathogens (e.g., bacteria and viruses) are inactivated within minutes in water with 1 ppm free chlorine when the pH is 7.2–7.8 and temperature is 77°F (25°C). CDC recommends, and jurisdictions typically require, a minimum of 1 ppm free chlorine in treated recreational water venues open to the public, including splash pads. However, maintaining an adequate free chlorine concentration can be especially difficult in splash pads because spraying or jetting the water aerosolizes free chlorine, which decreases its concentration. Oxidizing organic and nitrogenous compounds introduced from the environment (e.g., on splash pad users’ shoes) or by the splash pad users themselves (e.g., feces and urine) also deplete the free chlorine concentration. An analysis of inspection data from 16 jurisdictions in five states found 10% of routine inspections of splash pads identified violations related to improper disinfection levels (i.e., disinfectant concentration was too low or too high) (*13*). A Tennessee water quality survey of splash pads found samples from five (21%) of 24 splash pads had <1 ppm free chlorine (*10*). Not maintaining an adequate free chlorine concentration increases the risk for transmission of pathogens readily inactivated by free chlorine.

In contrast, *Cryptosporidium* oocysts, the infectious life stage of the parasite, are tolerant to chlorine. Oocysts can survive for >7 days in water with 1 ppm free chlorine when the temperature is 77°F (25°C) and the pH is 7.2–7.8 (*14*,*15*). The pH determines the amount of hypochlorous acid, the most active disinfectant form of chlorine also referred to as free chlorine, relative to the amount of hypochlorite ion, a less active disinfectant form of chlorine. A pH of 7.2–7.8 strikes a balance between maximizing free chlorine and minimizing swimmer discomfort and equipment corrosion. Temperature determines the rate of pathogen inactivation. The extreme chlorine tolerance of oocysts allows for *Cryptosporidium* transmission in splash pad water even with an adequate free chlorine concentration, enabling *Cryptosporidium* to cause larger outbreaks than those caused by pathogens readily inactivated by free chlorine (*16*). *C. hominis* caused 23% of the outbreaks overall and of those associated with splash pads only. These outbreaks resulted in 74% of cases overall and 85% of cases resulting from outbreaks associated with splash pads only. Because additional outbreaks were caused by *Cryptosporidium* of unknown species, the counts of outbreaks caused by *C. hominis* and of resulting cases are likely higher. Identifying *C. hominis* as the outbreak etiology indicates a human source of contamination and underscores the need to engage caregivers of young splash pad users because other species of *Cryptosporidium* might indicate fecal contamination from animals (e.g., squirrels, birds, and raccoons).

Overall, maintenance-related contributing factors were the most frequently reported (including both documented/observed and suspected factors) for outbreaks associated with splash pads only. These factors primarily focus on failures of disinfection systems. However, for splash pad only outbreaks caused by *Cryptosporidium*, person-related contributing factors were most frequently reported. This evidence suggests that improvements in recommended splash pad use, especially by young children, could help reduce incidence of splash pad–associated outbreaks caused by the most common etiological agent, *Cryptosporidium.* This evidence also is consistent with *Cryptosporidium* being tolerant to primary chemical disinfectants (e.g., chlorine). Conversely, inadequate water quality monitoring and testing policies were the second most frequently reported contributing factor for bacteria and viruses, following disinfectant system failures, suggesting jurisdictions having code requirements for water quality monitoring and properly designed, maintained, and operated disinfection systems would have a large impact on reducing splash pad–associated outbreaks caused by chemically susceptible etiologic agents.

CDC’s 2023 Model Aquatic Health Code (MAHC), developed collaboratively by public health officials, representatives of the aquatics sector, and researchers, is a set of design, construction, operation, and management recommendations to prevent illness and injury associated with treated recreational water venues open to the public (https://www.cdc.gov/model-aquatic-health-code/php/our-work/index.html). The 2023 MAHC addresses splash pads in which water is recirculated but not single-pass splash pads. MAHC splash pad design and construction recommendations include using secondary treatment, defined as reducing *Cryptosporidium* oocysts. For example, the use of ultraviolet light or ozone (*17**,**18*) by 3-log_10_ (99.9%) in splash pads but only 2-log_10_ (99%) in all other treated recreational water venues (e.g., pools) (MAHC section 4.7.3.3.2.1). This recommendation addresses the contributing factor of “no supplemental disinfection installed that would have inactivated the pathogen (e.g., *Cryptosporidium*)” (Table 4). Both the terms “secondary disinfection” and “supplemental disinfection” refer to inactivating *Cryptosporidium*. MAHC also recommends that secondary treatment systems treat 100% of splash pad water but only a portion (up to 100%) of the water in all other treated recreational water venues (MAHC section 4.7.3.3.2.2). These two recommendations are more stringent for splash pads because splash pads are at increased risk for contamination by *Cryptosporidium*.

MAHC operation recommendations for all venues (e.g., splash pads and pools) include maintaining a minimum of 1 ppm free chlorine (MAHC section 5.7.3.1.1.2) at pH 7.0–7.8 (MAHC section 5.7.3.4.1), monitoring free chlorine concentration and pH every 2–4 hours (MAHC sections 5.7.5.2–5.7.5.3), and using a proper test to measure free chlorine concentration and pH (MAHC sections 4.7.3.5.1 and 5.7.3.6.1–5.7.3.6.1.1). Following these recommendations enables splash pad operators to detect problems with disinfection systems. MAHC splash pad management recommendations include having a qualified operator onsite or immediately available within 2 hours during operating hours (MAHC sections 6.3.1.1.1–6.3.1.1.3) so that venues are operated and managed to minimize the risk for illness and injury (e.g., maintaining adequate concentration of free chlorine). Qualified operators are those who have successfully completed training recognized by the jurisdiction. Operator training decreases operator errors and has been associated with better operation and water quality in swimming pools (*13*).

## Limitations

The findings in this report are subject to at least three limitations. First, NORS data likely underestimate the actual occurrence of splash pad–associated outbreaks because of underreporting or misclassification. Outbreaks might not be reported at all, because of the lengthy incubation period of *Cryptosporidium* (up to nearly 2 weeks), lack of regulatory authority by public health personnel over splash pads in certain jurisdictions, differences in investigation and reporting capacity among health departments, and the voluntary nature of reporting outbreaks to CDC, all of which might present barriers to the detection, investigation, and reporting of splash pad–associated outbreaks. Moreover, the public health response to the COVID-19 pandemic was resource and time intensive, which could have been an additional barrier to 2020–2022 outbreak investigation, reporting, and subsequent efforts to finalize data on outbreaks that started in 2018 or later (*6*). In addition, splash pad or outbreak source might be misclassified as another venue type. For example, an outbreak associated with a splash pad in a wildlife park was reported to NORS but was listed as a water park venue and not a splash pad (*5*). Second, investigation and reporting of recreational waterborne disease outbreaks with a chemical etiology might be limited because of the potentially transient nature of chemical contamination and potential lack of communication between those who respond to these outbreaks (e.g., hazardous materials personnel) and the infectious disease epidemiologists, who report outbreaks to CDC. Finally, the representativeness of NORS data on factors contributing to splash pad–associated outbreaks could be limited. Environmental health specialists primarily identify contributing factors by conducting inspections of regulated venues implicated in outbreak investigations, but infectious disease epidemiologists report the outbreaks to CDC. The different roles, and often different governmental units, of environmental health specialists and infectious disease epidemiologists within jurisdictions might require additional effort for collaboration, communication, and reporting data on contributing factors.

## Future Directions

Prevention of waterborne disease outbreaks at splash pads requires changes in user behavior; recreational venue code updates; and improved venue design, construction, operation, and management of facilities. Recommended user behaviors in splash pads and other recreational water venues include not getting in the water if ill with diarrhea until two weeks after it has stopped, not swallowing the water, taking young children on bathroom breaks or checking diapers or swim diapers every hour, and, if needed, changing them away from the water (https://www.cdc.gov/healthy-swimming/prevention/index.html). A recommended splash pad–specific behavior is not standing or sitting above the splash pad jets (*1*). Public health and the aquatics sector can partner to increase the public’s awareness of healthy splash pad use and to encourage these behaviors at splash pads and other recreational water venues through staff members’ action and signage. Because splash pads are intended for young children, these efforts need to be oriented toward and reach caregivers of young children. Jurisdictions can voluntarily update and revise their public health regulations using MAHC, to help prevent splash pad–associated outbreaks, especially those caused by *Cryptosporidium*. The aquatics sector also can collaborate with jurisdictions to voluntarily adopt MAHC recommendations. MAHC could be revised to include single-pass splash pads. In 2023, CDC revised NORS (Form 52.14) to improve data quality by streamlining and updating data collection fields (https://www.cdc.gov/nors/pdf/NORS-Form-CDC-52.14_508.pdf). This revision included substantially reducing the number of listed contributing factors to simplify reporting. NORS can be further revised to collect data so that splash pads in which water is recirculated and single-pass splash pads can be distinguished. Comprehensive data describing outbreaks can be used to help optimize the development of prevention strategies. CDC recommends epidemiologists, environmental health specialists, microbiologists, communicators, and behavioral scientists collaborate, as needed, on investigations of waterborne outbreaks associated with splash pads and other recreational water venues to control and characterize outbreaks.

## Conclusion

Recreational water activities promote social and health benefits by providing cooling relief from heat and splash pads represent a reduced risk for drowning for users compared with swimming pools and other aquatic venues. Although splash pads can create an opportunity for pathogen transmission, particularly to young children, outbreaks associated with splash pads are preventable. Public health officials and the aquatics sector can use the findings in this report to promote the prevention of splash pad–associated outbreaks (e.g., recommended user behaviors) and guide the construction, operation, and management of splash pads.

## Footnotes

* 45 C.F.R. part 46.102(l)(2), 21 C.F.R. part 56; 42 U.S.C. Sect. 241(d); 5 U.S.C. Sect. 552a; 44 U.S.C. Sect. 3501 et seq.
